# Impact of the COVID-19 pandemic on incidence and severity of acute appendicitis: a comparison between 2019 and 2020

**DOI:** 10.1186/s12873-021-00454-y

**Published:** 2021-05-12

**Authors:** Jochem C. G. Scheijmans, Alexander B. J. Borgstein, Carl A. J. Puylaert, Wouter J. Bom, Said Bachiri, Eduard A. van Bodegraven, Amarins T. A. Brandsma, Floor M. ter Brugge, Steve M. M. de Castro, Roy Couvreur, Lotte C. Franken, Marcia P. Gaspersz, Michelle R. de Graaff, Hannah Groenen, Suzanne C. Kleipool, Toon J. L. Kuypers, Milou H. Martens, David M. Mens, Ricardo G. Orsini, Nando J. M. M. Reneerkens, Thomas Schok, Wouter J. A. Sedee, Shahzad Tavakoli Rad, José H. Volders, Pepijn D. Weeder, Jan M. Prins, Hester A. Gietema, Jaap Stoker, Suzanne S. Gisbertz, Marc G. H. Besselink, Marja A. Boermeester, the SCOUT Collaboarative Study group, C. S. Andeweg, E. G. Boerma, M. D. M. Bolmers, M. J. Bolster van-Eenennaam, L. S. F. Boogerd, P. van Duijvendijk, S. L. Gans, A. A. W. van Geloven, P. D. Gobardhan, E. R. Hendriks, J. T. ten Holder, J. M. Hoogendoorn, T. de Hoop, J. van Kesteren, J. L. M. Konsten, S. Kucukcelebi, A. M. F. Lopes Cardozo, G. M. H. Marres, R. A. Matthijsen, A. W. F. du Mée, S. van der Meij, J. Melenhorst, F. H. M. van Osch, G. A. Patijn, R. A. W. Ploumen, T. J. M. Quanjel, T. F. D. van Rees Vellinga, M. A. J. de Roos, C. C. van Rossem, H. M. E. Quarles van Ufford, H. C. van Santvoort, N. Sosef, D. Schweitzer, T. Verhagen

**Affiliations:** 1 Department of Surgery, Amsterdam UMC, location AMC, Amstserdam Gastroenterology Endocrinology Metabolism, University of Amsterdam, Meibergdreef 9, 1105 AZ Amsterdam, the Netherlands; 2 Department of Surgery, Cancer Center Amsterdam, Amsterdam UMC, University of Amsterdam, Amsterdam, the Netherlands; 3 Department of Radiology and Nuclear Medicine, Amsterdam Gastroenterology Endocrinology Metabolism, Amsterdam UMC, University of Amsterdam, Amsterdam, the Netherlands; 4 Department of Surgery, Noordwest Hospital Group, Alkmaar, the Netherlands; 5 Department of Surgery, Albert Schweitzer Hospital, Dordrecht, the Netherlands; 6 Department of Surgery, Isala Hospital, Zwolle, the Netherlands; 7 Department of Surgery, Hospital Group Twente, Almelo, the Netherlands; 8 Department of Surgery, OLVG, Amsterdam, the Netherlands; 9 Department of Surgery, Haaglanden Medical Center, The Hague, the Netherlands; 10 Departement of Surgery, Flevo Hospital, Almere, the Netherlands; 11 Department of Surgery, Maasstad Hospital, Rotterdam, the Netherlands; 12 Department of Surgery, Gelre Hospitals, Apeldoorn, the Netherlands; 13 Department of Surgery, Tergooi Hospitals, Hilversum, the Netherlands; 14 Department of Surgery, Elisabeth - Tweesteden Hospital, Tilburg, the Netherlands; 15 Department of Surgery, Zuyderland Medical Center, Sittard-Geleen/Heerlen, the Netherlands; 16 Department of Surgery, Amphia Hospital, Breda, the Netherlands; 17 Department of Surgery, Maastricht UMC+, Maastricht, the Netherlands; 18 Department of Surgery, Dijklander Hospital, Hoorn, the Netherlands; 19 Department of Surgery, VieCuri Medisch Centrum for Noord-Limburg, Venlo, the Netherlands; 20 Department of Emergency Medicine, St Jansdal Hospital, Harderwijk, the Netherlands; 21 Department of Surgery, Sint Antonius Hospital, Nieuwegein, the Netherlands; 22 Department of Surgery, Rijnstate Hospital, Arnhem, the Netherlands; 23 Department of Surgery, Spaarne Gasthuis, Haarlem, and Hoofddorp, the Netherlands; 24 Department of Internal Medicine, Division of Infectious Diseases, Amsterdam Institute for Infection and Immunity (AI&II), Amsterdam UMC, University of Amsterdam, Amsterdam, the Netherlands; 25 Department of Radiology and Nuclear Medicine, Maastricht UMC+, Maastricht, the Netherlands

**Keywords:** Acute appendicitis, COVID-19 pandemic, Complicated appendicitis

## Abstract

**Background:**

During the COVID-19 pandemic, a decrease in the number of patients presenting with acute appendicitis was observed. It is unclear whether this caused a shift towards more complicated cases of acute appendicitis. We compared a cohort of patients diagnosed with acute appendicitis during the 2020 COVID-19 pandemic with a 2019 control cohort.

**Methods:**

We retrospectively included consecutive adult patients in 21 hospitals presenting with acute appendicitis in a COVID-19 pandemic cohort (March 15 – April 30, 2020) and a control cohort (March 15 – April 30, 2019). Primary outcome was the proportion of complicated appendicitis. Secondary outcomes included prehospital delay, appendicitis severity, and postoperative complication rates.

**Results:**

The COVID-19 pandemic cohort comprised 607 patients vs. 642 patients in the control cohort. During the COVID-19 pandemic, a higher proportion of complicated appendicitis was seen (46.9% vs. 38.5%; *p* = 0.003). More patients had symptoms exceeding 24 h (61.1% vs. 56.2%, respectively, *p* = 0.048). After correction for prehospital delay, presentation during the first wave of the COVID-19 pandemic was still associated with a higher rate of complicated appendicitis. Patients presenting > 24 h after onset of symptoms during the COVID-19 pandemic were older (median 45 vs. 37 years; *p* = 0.001) and had more postoperative complications (15.3% vs. 6.7%; *p* = 0.002).

**Conclusions:**

Although the incidence of acute appendicitis was slightly lower during the first wave of the 2020 COVID-19 pandemic, more patients presented with a delay and with complicated appendicitis than in a corresponding period in 2019. Spontaneous resolution of mild appendicitis may have contributed to the increased proportion of patients with complicated appendicitis. Late presenting patients were older and experienced more postoperative complications compared to the control cohort.

**Supplementary Information:**

The online version contains supplementary material available at 10.1186/s12873-021-00454-y.

## Introduction

The first wave of the 2020 COVID-19 pandemic resulted in a reduction of acute care surgeries [1]. Lockdown measures, patients’ fear of contracting COVID-19 during hospital visits, and reluctance to burden the overloaded healthcare system by requesting care for non-COVID complaints, may have led to a higher threshold for seeking medical care.

For appendicitis, an increased prehospital delay during the pandemic has been reported [2, 3]. Also, a shift towards a higher proportion of complicated appendicitis cases has been described, both in adults and children [2–6], as well as a decrease in the total number of patients compared to the weeks prior to COVID [7, 8]. Although all previous studies show the same shift towards relatively more complicated appendicitis patients, the cohorts were small and control groups were insufficient. Patients with uncomplicated appendicitis may have stayed at home and recovered spontaneously [7, 8], which would support the theory that uncomplicated and complicated appendicitis are different diseases and not simply different grades of severity [9–11].

A recent meta-analysis in the pre-COVID era showed that delayed appendectomy up to 24 h in patients with presumed uncomplicated appendicitis does not increase the risk for developing complicated appendicitis [12]. Moreover, conservative treatment with antibiotics has been proven to be as safe and effective as surgical treatment in patients with uncomplicated appendicitis [13, 14]. Some uncomplicated appendicitis may indeed resolve spontaneously [9, 10, 15, 16].

The present study aims to compare the proportions of uncomplicated and complicated appendicitis in adult patients presenting during the first wave of the COVID-19 pandemic with a control cohort from the corresponding time period in 2019.

## Methods

This retrospective multicenter study was conducted at two academic and 19 non-academic hospitals in the Netherlands. Ethical approval was waived by a central institutional review board because of the observational nature of the study. This decision was endorsed by the institutional review board of each participating center. Permission of patient participation was obtained through an opt-out procedure, as was customary for COVID-19 related observational research.

### Study population

Consecutive adult patients (≥ 18 years) presenting with a diagnosis of acute appendicitis were included in two cohorts. The pandemic cohort included patients presenting between March 15 and April 30, 2020, immediately after the start of the COVID-preventing semi-lockdown measures in the Netherlands. The pre-pandemic control cohort included patients presenting in the corresponding period in 2019, between March 15 and April 30. Patients were identified by searching the electronic patient file databases via ICD-10 codes (appendicitis, acute abdominal pain, peritonitis or intra-abdominal abscesses) and searching emergency department (ED) patient lists and surgery lists from that period. Patients were included if the final diagnosis was acute appendicitis. No formal sample size calculation was performed, but a fixed inclusion period was set. Post hoc power analysis was executed based on the proportion of patients with complicated appendicitis.

### Data collection

Patient demographics, comorbidities, clinical and imaging data from the ED, information about treatment modality, operation notes, pathology results, and 30-day clinical follow-up were collected from electronic patient records. Times of arrival at the ED and start of treatment were also retrieved, as well as imaging diagnoses. If patients were operated within 30 days after initially being treated conservatively with antibiotics, 30-day postoperative follow-up was collected. Participants were pseudonymized to ensure patient’s privacy.

### Definitions

Appendicitis severity was determined according to the operation notes and pathology reports, or imaging reports in cases of conservative treatment. In those cases where more than one imaging modality was used, reports of the imaging modality that confirmed the diagnosis of appendicitis were used.

Uncomplicated appendicitis was defined as an inflamed appendix or periappendicitis without signs of necrosis or perforation as described by surgeon and pathologist. Complicated appendicitis was defined as inflammation of the appendix with presence of gangrene, evident necrosis or perforation, as described by the pathologist, and/or presence of perforation or abscess formation, as described by the surgeon. Conservatively treated patients with a periappendicular abscess or widespread infiltration on imaging, were also scored as complicated. If the pathologist found a normal appendix, the latter overruled the diagnosis of the surgeon. In the few cases where no histological analysis was performed, e.g. because of full necrosis of the appendix, the diagnosis as established during surgery was used.

Conservative treatment was defined as initial treatment with antibiotics and/or percutaneous drainage of a periappendicular abscess. In operated patients, the in-hospital delay or time to surgery was defined as the time between presentation at the ED and the start of the operation. Postoperative complications were scored according to the Clavien-Dindo scale [17]. Complications scored as III or higher were defined as severe complications.

### Study outcomes

The primary outcome of this study was the difference in appendicitis severity distribution (i.e., the proportion of patients presenting with complicated appendicitis) between the COVID-19 pandemic cohort (2020) and the control cohort (2019). Secondary outcomes were the differences in baseline characteristics, pre- and in-hospital delay, number of perforated appendicitis, type of treatment, postoperative complications, complications in general, and the daily rate of patients presenting with acute appendicitis between both cohorts.

### Statistical analysis

The cohorts were compared and stratified for appendicitis severity and duration of symptoms. This duration was dichotomized by visual analysis of a chart of the duration of symptoms at presentation, see supplements. Confidence intervals for proportions were calculated by the Wilson score method without continuity correction. Univariate analyses were performed using the Chi-squared or Fisher’s exact test for categorical variables, and the Mann-Whitney U test for continuous variables. To quantify the possible association between presentation during the COVID-19 pandemic and having complicated appendicitis, a multivariable logistic regression analysis was performed, adjusted for duration of symptoms longer than 24 h. A post hoc power analysis was conducted. All *P* values were based on two-sided tests and *P* < 0.05 was considered statistically significant. Missing data were not imputed, but described as missing. Data were analyzed using SPSS for Windows version 26 (IBM, Armonk, New York, USA).

## Results

**Fig. 1:**
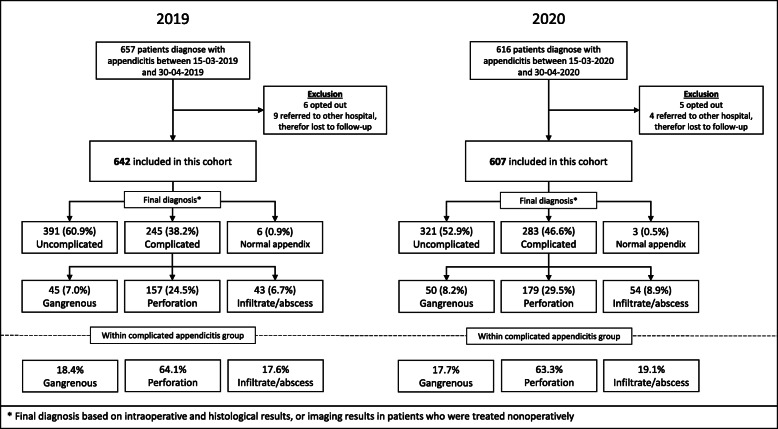
Flowchart: inclusions SCOUT-4 study cohort versus 2020

**Fig. 2:**
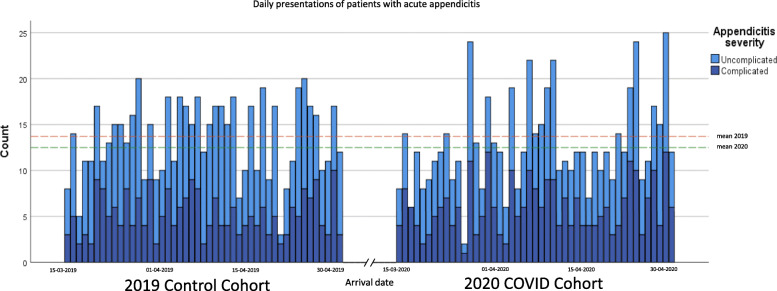
Daily presentations of patients with acute appendicitis

Between March 15 and April 30, 607 out of 616 eligible patients with acute appendicitis were included in the pandemic cohort (2020) and 642 of 657 eligible patients in the control cohort (2019), see Fig. 1. Only 1.5 and 2.3% of eligible patients were excluded from the pandemic and control cohort, respectively. During the COVID-19 pandemic, an absolute decrease of 5.5% (95% C.I. 4.0–7.5%) in the numbers of patients diagnosed with appendicitis was seen as compared to 2019; this was a 6.2% (95% C.I. 4.6–8.3%) decrease in all eligible patients (Fig. 1). The mean daily presentation rate was constant over time in both cohorts (Fig. 2). Demographic and clinical characteristics of patients in both cohorts are presented in Table 1. Comorbidities such as diabetes and coronary artery disease were somewhat more common in the pandemic cohort. However, no significant differences in comorbidities among cohorts were seen. All patients underwent diagnostic imaging before treatment. In 2020, more patients were diagnosed by CT (and not ultrasound (US)) compared to 2019 (51.9% vs. 34.9%, *p* < 0.001) because of COVID-related restricted use of US.

**Table 1:** Baseline: clinical characteristics of all patients, 2019 pre-COVID cohort compared to 2020 COVID cohort

Characteristic	2019 control cohort (*n* = 642)	2020 COVID-19 cohort (*n* = 607)	*P* value
Age, median (IQR), years	40 (28–57)	42 (29–58)	0.183
Female sex, no./total no. (%)	322/642 (50.2)	318/607 (52.4)	0.430
ASA > 1, no./total no. (%)	263/595 (44.2)	254/524 (48.5)	0.153
COPD, no./total no. (%)	13/630 (2.1)	11/599 (1.8)	0.774
Diabetes Mellitus, no./total no. (%)	24/631 (3.8)	34/596 (5.7)	0.117
Heart failure, no./total no. (%)	11/631 (1.7)	11/597 (1.8)	0.896
Coronary artery disease, no./total no. (%)	17/631 (2.7)	28/598 (4.7)	0.064
Active smoker, no./total no. (%)	61/293 (20.8)	63/275 (22.9)	0.547
Duration of symptoms > 24 h, no./total no. (%)	358/637 (56.2)	371/601 (61.1)	**0.048**
Severity of appendicitis, no./total no. (%)			**0.008**
Uncomplicated	391/642 (60.9)	321/607 (52.9)	
Gangrenous	45/642 (7.0)	50/607 (8.2)	
Perforation	157/642 (24.5)	179/607 (29.5)	
Infiltrate/Abscess	43/642 (6.7)	54/607 (8.9)	
Normal (sana)	6/642 (0.9)	3/607 (0.5)	
Conservative treatment, no./total no. (%)	41/642 (6.4)	63/607 (10.4)	**0.011**
Complication within 30 days, no./total no. (%)	72/642 (11.5)	76/607 (12.5)	0.475

*Abbreviations*: *ASA* American Society of Anesthesiologists, *COPD* Chronic Obstructive Pulmonary Disease, *IQR* interquartile range

### Appendicitis severity

**Fig. 3:**
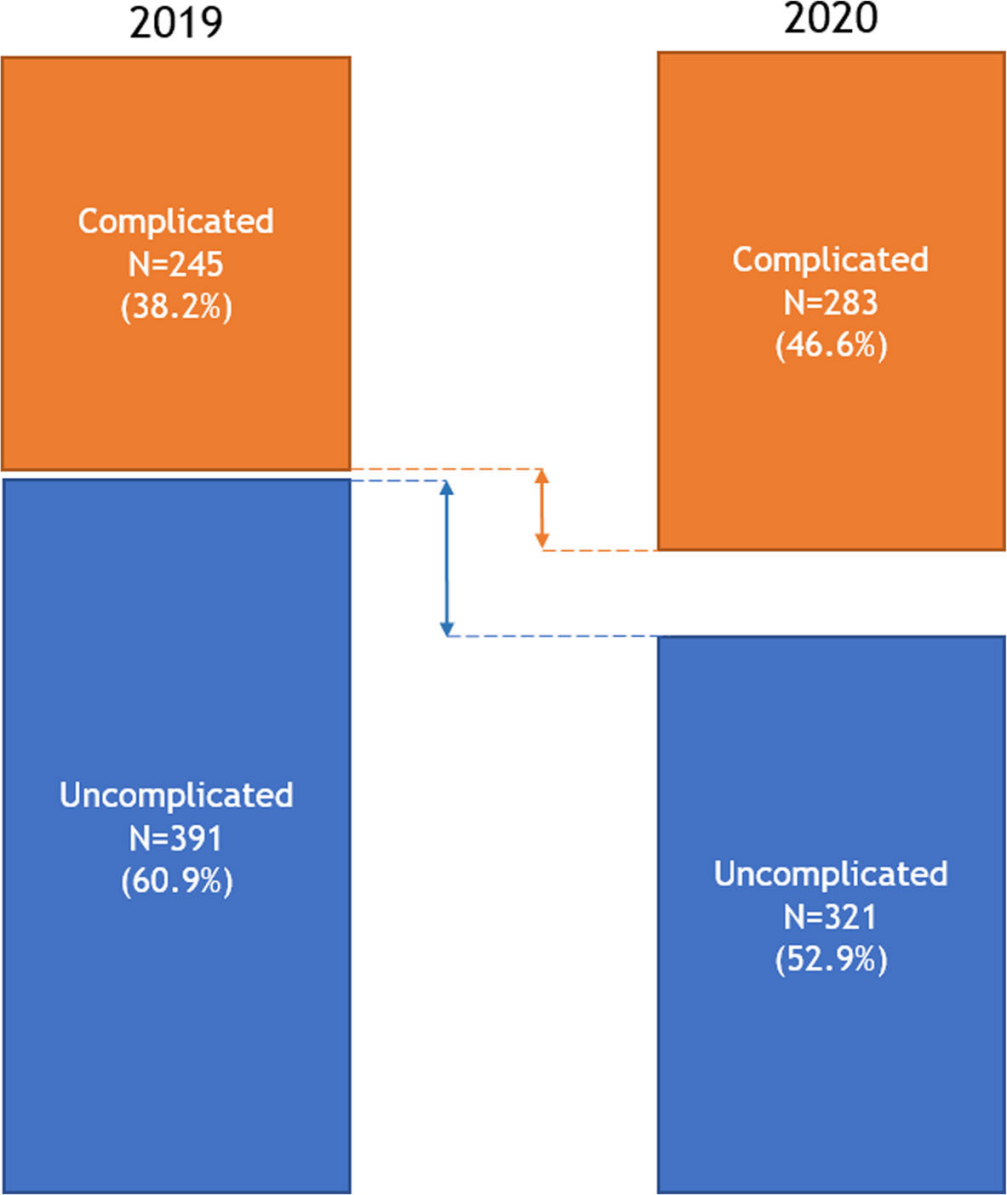
Total number of patients, stratified by type of acute appendicitis: 2019 control cohort vs. 2020 COVID cohort

As shown in Table 1 and Fig. 3, in the 2020 pandemic cohort a higher proportion of patients presented with complicated appendicitis compared to the 2019 control cohort (46.6% vs. 38.2%, respectively, *p* = 0.008). The perforation rate in the 2020 cohort was 29.5% versus 24.5% in the control cohort (*p* = 0.045). Focusing only on complicated appendicitis patients, no differences in perforation rate (63.3% vs. 64.1%, *p* = 0.84) or rate of periappendiculair infiltrate/abscess formation (19.1% vs. 17.6%, *p* = 0.65) were found between the 2020 and 2019 cohorts (Table 2).

**Table 2:** Comparison of patients with complicated appendicitis for 2019 pre-COVID cohort vs. 2020 COVID cohort

Characteristic	2019 control cohort (*n* = 245)	2020 COVID-19 cohort (*n* = 283)	*P* value
Age, median (IQR), years	49 (33–65)	50 (32–64)	0.946
ASA > 1, no./total no. (%)	126/225 (56.0)	131/239 (54.8)	0.797
Duration of symptoms > 24 h, no./total no. (%)	164/241 (68.0)	214/281 (76.2)	**0.039**
Types of complicated appendicitis, no./total no. (%)			
Gangrenous	45/245 (18.4)	50/283 (17.7)	0.835
Perforation	157/245 (64.1)	179/283 (63.3)	0.843
Abscess or infiltrate	43/245 (17.6)	54/283 (19.1)	0.651
Conservative treatment, no./total no. (%)	31/245 (12.7)	41/283 (14.5)	0.540
In-hospital delay in operated patients, median (IQR), hours	7.8 (5.0–13.3)	6.7 (4.6–12.1)	0.152
Postoperative complication^a^, no./total no. (%)	40/214 (18.7)	45/242 (18.6)	0.979
Severe postoperative complication^a b^, no./total no. (%)	11/245 (5.1)	13/281 (5.4)	0.904

*Abbreviations*: *ASA* American Society of Anesthesiologists, *IQR* interquartile range

^a^Patients for whom surgery was the initial treatment

^b^Severe complications are defined as Clavien-Dindo IIIa or higher

### Duration of symptoms

In the 2020 pandemic cohort, relatively more patients presented at the hospital with symptoms present for > 24 h compared to the control cohort (61.1% vs. 56.2%, *p* = 0.048; Table 1). In the group of patients with complicated appendicitis, more late presentations were seen in the pandemic group than in the 2019 control group (76.2% vs. 68.0%, *p* = 0.039; Table 2). This difference was not seen in patients with uncomplicated appendicitis (51.5% vs. 51.4%, *p* = 0.98; Table S1).

In Table 3, patients are stratified according to duration of symptoms at presentation; ≤24 h or > 24 h. During the COVID-19 pandemic, patients presenting after > 24 h were older than patients presenting within 24 h after onset of symptoms (median 45 years (31–60) vs. 37 years (28–52); *p* = 0.001). In the control group, no age difference was seen in time of presentation. Additionally, a larger proportion of patients with an increased risk for a more severe course of COVID-19 (age ≥ 60 years) presented after > 24 h during the COVID-19 pandemic compared to the control 2019 cohort (72.2% vs 60.3%, *p* = 0.039; Table S3). Patients with complicated appendicitis and symptoms for > 24 h had a comparable perforation rate in both cohorts (64.0% vs. 66.5%, *p* = 0.62; see supplementary Table S2).

**Table 3:** Characteristics and outcomes of patients with appendicitis, stratified for duration of symptoms, ≤24 h vs. > 24 h, in 2019 pre-COVID cohort and 2020 COVID cohort

Characteristic	2019 control cohort (*n* = 637)^b^		*P* value	2020 COVID-19 cohort (*n* = 601)^b^		*P* value
	≤ 24 h (*n* = 279)	> 24 h (*n* = 358)		≤ 24 h (*n* = 230)	> 24 h (*n* = 371)	
Age, median (IQR), years	40 (28–55)	41 (27–58)	0.707	37 (28–52)	45 (31–60)	**0.001**
Age ≥ 60 years, no./total no. (%)	54/279 (19.4)	82/358 (22.9)	0.278	37/230 (16.1)	96/371 (25.9)	**0.005**
Female sex, no./total no. (%)	151/279 (54.1)	169/358 (47.2)	0.083	124/230 (53.9)	189/371 (50.9)	0.479
ASA > 1, no./total no. (%)	115/263 (43.7)	145/327 (44.3)	0.881	106/211 (50.2)	145/309 (46.9)	0.458
Comorbidity, no./total no. (%)	22/273 (8.1)	24/348 (6.9)	0.583	20/223 (9.0)	44/363 (12.1)	0.235
Severity of appendicitis, no./total no. (%)			**< 0.001**^a^			**< 0.001**^a^
Uncomplicated	201/279 (72.0)	189/358 (52.8)		163/230 (70.9)	154/371 (41.5)	
Gangrenous	26/279 (9.3)	19/358 (5.3)		17/230 (7.4)	32/371 (8.6)	
Perforation	44/279 (15.8)	109/358 (30.4)		41/230 (17.8)	137/371 (36.9)	
Infiltration/abscess	7/279 (2.5)	36/358 (10.1)		9/230 (3.9)	45/371 (12.1)	
Normal (sana)	1/279 (0.4)	5/358 (1.4)		0/230 (0)	3/371 (0.8)	
Conservative treatment, no./total no. (%)	8/279 (2.9)	33/358 (9.2)	**0.001**	5/230 (2.2)	57/371 (15.4)	**< 0.001**^a^
In-hospital delay in operated patients, median (IQR), hours	7.6 (5.0–14.7)	7.0 (4.8–11.8)	0.076	7.2 (4.4–13.2)	6.2 (4.3–10.0)	0.057
Complication within 30 days, no./total no. (%)	28/279 (10.0)	42/358 (11.7)	0.292	16/230 (7.0)	60/371 (16.2)	**0.001**
Postoperative complication^c^, no./total no. (%)	26/271 (9.6)	41/325 (12.6)	0.245	15/225 (6.7)	48/313 (15.3)	**0.002**
Severe postoperative complication^c d^, no./total no. (%)	4/271 (1.5)	10/325 (3.1)	0.279^a^	5/225 (2.2)	15/313 (4.8)	0.165^a^

*Abbreviations*: *ASA* American Society of Anesthesiologists, *IQR* interquartile range

^a^Fischer exact test was performed

^b^In 2019 5 patients and in 2020 6 patients missed data about the duration of symptoms

^c^Patients for whom surgery was the initial treatment

^d^Severe complications are defined as Clavien-Dindo IIIa or higher

In a univariate logistic regression analysis, the odds ratios (ORs) for complicated appendicitis were 1.41 (95% CI: 1.12–1.76) for patients presenting during the COVID-19 pandemic and 2.79 (95% CI: 2.12–3.55) for patients with symptoms for more than 24 h. In multivariate logistic regression including both variables, these associations persisted, with ORs of 1.38 (95% CI: 1.10–1.75) for presentation during the COVID-19 pandemic and 2.75 (95% CI: 2.16–3.51) for duration of symptoms > 24 h, respectively.

### Initial treatment

During the COVID-19 pandemic, 544 (89.6%) patients underwent surgery compared to 601 (93.6%) patients in the 2019 control cohort (*p* = 0.011). Of these patients, 532 (97.8%) and 587 (97.7%) were operated laparoscopically. In respectively five (0.9%) and ten (1.7%) cases, the procedure was converted to open appendectomy. The median in-hospital time to surgery during the COVID-19 pandemic was 6.5 (4.3–11.8) hours, which was shorter than the 7.4 (4.9–13.6) hours in the 2019 control cohort (*p* = 0.004). In the 2020 cohort, 63 (10.4%) patients were initially treated conservatively versus 41 (6.4%) in the 2019 control cohort (*p* = 0.011), see Tables 1 and S4. The majority of these patients were diagnosed with complicated appendicitis (65.1% in 2020 and 75.6% in 2019). Within the 2020 pandemic cohort, a higher proportion of conservatively treated patients were diagnosed with complicated appendicitis by use of initial CT compared to the 2019 control cohort, see Table S5.

### Complications

No differences were found in number of postoperative complications between the COVID-19 cohort and the control cohort. However, in patients presenting during the COVID-19 pandemic, more complications were seen in patients presenting with symptoms for more than 24 h compared to patients who present earlier (16.2% vs. 7.0%; *p* = 0.001). This difference was not found within the 2019 control cohort (Table 3). Within the 2020 cohort, 12 patients tested positive for COVID-19. Eleven were confirmed by RT-PCR and one was diagnosed based on chest CT (Table S6).

### Power analysis

Post hoc power analysis was performed. The cohort sizes of 642 and 607 patients, proportions of patients with complicated appendicitis of 46.6 and 38.2% and an α of 0.05 resulted in a power of 85.2%, which was considered as being sufficient.

## Discussion

This large multicenter study compared adult patients presenting with acute appendicitis during the COVID-19 pandemic with patients presenting in the corresponding period of the pre-COVID year 2019. Although only a slight decrease of patients presenting with acute appendicitis was observed during the first COVID-19 wave compared to 2019, a higher proportion presented with complicated appendicitis. The perforation rate among patients with complicated appendicitis, however, was unaffected. During the COVID-19 pandemic, more patients presented with a prehospital delay of more than 24 h. These patients were older and endured more postoperative complications compared to patients presenting with symptoms for less than 24 h. This association was not found in the 2019 control cohort.

Present data suggest an association between prehospital delay and complicated appendicitis. This is in line with previous, small studies describing higher proportions of complicated appendicitis during the COVID-19 pandemic. Dreifuss et al. found complicated appendicitis in seven (46.7%) out of 15 adult Argentinian patients with acute appendicitis during April 2020 compared to 11 (16.9%) out of 65 patients during April 2018 and 2019 [3]. Patients in the 2020 cohort show a longer delay in presentation than the control group (58.4 vs. 32.8 h) [3]. These differences are confirmed by Gao et al., who analyzed a Chinese cohort of 163 patients who presented with appendicitis between June 2019 and April 2020 [2]. They find complicated appendicitis in 51.7% of patients and a mean prehospital delay of 65.0 h in the epidemic cohort (presentation after January 1st), compared to 12.4% complicated appendicitis and a mean delay of 17.3 h in the pre-epidemic cohort (both *p* < 0.001) [2]. Gao et al. show a significant increase in requests for conservative treatment during the COVID-19 outbreak [2]. Both increased prehospital delay and reduced willingness to be operated may be explained by fear of contracting SARS-CoV-2 in hospitals [2]. Our data showed a longer prehospital delay during the pandemic and a significant higher age in patients who presented more than 24 h after onset of symptoms. Fear of a SARS-CoV-2 infection could have caused this delay particularly in older patients, as those patients have an intrinsic higher risk for a more severe course of COVID-19 [18]. This may have resulted in some form of inclusion bias, because mild cases may have resolved spontaneously by refraining from consultation with a doctor.

Multivariable regression analysis showed an association between complicated appendicitis and presentation during the first wave of the COVID-19 pandemic, independent of late presentation. This implies that another factor could have influenced the appendicitis severity during the pandemic. An Israeli study showed a significant decrease in patients admitted with uncomplicated appendicitis during the first weeks since the onset of COVID-19, compared to an antecedent period; 204 uncomplicated appendicitis cases pre-pandemic compared to 111 during the pandemic. The number of complicated cases and the prehospital delay in both cohorts were comparable [7]. Neufeld et al. also describe a significant decrease of the number of presented uncomplicated appendicitis cases during the COVID-19 pandemic compared to the 2 years before [8]. The number of complicated cases in their multicenter cohort, consisting of 956 adult acute appendicitis patients, remained stable [8]. The authors of both studies hypothesized that the successful resolution of mild appendicitis at home could explain the decrease in total number of patients [7, 8]. In our 2020 COVID-19 cohort, a similar absolute decrease of the total number of uncomplicated appendicitis cases was found. Since the decrease of uncomplicated cases was greater than the increase of complicated cases, part of the patients with mild, uncomplicated appendicitis may have resolved spontaneously at home. This would be in line with epidemiological and clinical studies underlining two different entities of appendicitis [9–11] and the conclusion of Tankel and Neufeld et al. [7, 8]. However, the absolute decrease found in our study was relatively small compared to other studies such that ‘normal’ annual variability of acute appendicitis as a cause for the decrease cannot be ruled out. The difference between present study and the Israelian study could be explained by the lower mean age in the latter study (43 vs 23 years) [7]. Moreover, it may be concluded that the COVID-19 pandemic and the semi-lockdown measures in the Netherlands discouraged patients to visit an emergency department to a lesser extent than it did the Israelian patients during complete lockdown.

Significantly more patients were treated conservatively during the COVID-19 pandemic compared to the 2019 control cohort. However, the increase from 6.4 to 10.4% was much lower than the increase reported by the HAREM study group: adult patients presenting with acute appendicitis during the COVID-19 lock-down in the UK showed a more radical shift with 271 of 500 (54%) patients treated conservatively [19]. In this first report of the HAREM cohort no differentiation between uncomplicated and complicated appendicitis is provided and definitive conclusions have to wait until the final results become available [19]. Within our cohorts, most conservatively managed patients were cases of complicated appendicitis, receiving antibiotics with or without percutaneous drainage for a periappendiceal abscess, which is common practice [20]. More patients underwent initial CT during the pandemic, which may have resulted in better diagnosis of complicated cases and thereby more conservative treatment. In addition, we observed a limited increase of conservative treatment in uncomplicated cases (2.6 to 6.9%), which could also have been the result of the renewed Dutch national guideline (July 2019), stating that conservative treatment could be considered for uncomplicated appendicitis [21]. In the Netherlands, the national guideline was not changed to discourage the surgical treatment of acute appendicitis during the COVID-19 pandemic, which was done in some other countries, e.g. the UK. Therefore, the effect of the COVID-19 pandemic on the management of acute appendicitis in the Netherlands was minimal. Furthermore, the effect was predominantly explained by the shift towards more cases of complicated appendicitis.

Compared to previous large observational audits of acute appendicitis patients [22–24], a higher proportion of patients with complicated appendicitis was seen in both our pandemic and control cohorts. This difference may be caused by the definition we used for complicated appendicitis, which was based on the combined surgical and histological diagnoses, instead of only the surgical [22] or histological [23] diagnosis. Moreover, a low rate of normal appendices and the inclusion of conservatively treated patients, who were mostly diagnosed with complicated appendicitis in our cohorts, may have contributed to the discrepancy.

We found a significantly shorter in-hospital delay within the pandemic group. The median in-hospital time to surgery during the COVID-19 pandemic was 6.5 (4.3–11.8) hours, which was significantly shorter than the 7.4 (4.9–13.6) hours in the 2019 control cohort (*p* = 0.004). One could argue that the reason for this shorter in-hospital delay is due to the fact that patients had a more severe disease presentation as more patients in the pandemic cohort had complicated appendicitis and surgeons were therefore keener to operate quickly. However, we think that this difference was mainly influenced by logistic reasons. During the first wave of the COVID-19 pandemic, most of elective surgery was cancelled, resulting in more opportunities for immediate operations such as emergency appendectomies. Furthermore, it is unlikely that this shorter in-hospital delay compared to controls affected the number of complicated appendicitis cases. Complicated and uncomplicated appendicitis are most likely two different disease entities and, as illustrated by a recent meta-analysis of van Dijk et al., in-hospital delay up to 24 h does not lead to a higher rate of complicated appendicitis [12].

The findings of this study should be interpreted in light of some limitations. First, data were collected retrospectively and data were only available for patients who actually presented at the hospital. Therefore, the proportion of patients with complicated appendicitis within the total number of patients with acute appendicitis is most likely biased by the number of patients with a mild appendicitis not presenting in a hospital and who experienced spontaneous resolution of symptoms at home. Second, the semi-lockdown in the Netherlands was less strict compared to measures taken by other countries. This may have resulted in less impact of the COVID-19 pandemic on the presentation of patients with acute appendicitis compared to other countries such as Israel or Spain.

Strengths of this study are the large number of included patients and the multicenter cohort design. A control cohort from the corresponding time period in 2019 was included and no missing data were reported for primary outcomes. Another strength of this study is that a high proportion of patients in this cohort was treated surgically, resulting in confirmed diagnoses based on combined surgical and histological reports in the vast majority of patients. Finally, the inclusion period started at the moment of the national semi-lockdown in the Netherlands instead of starting after the first COVID-19 patient was diagnosed, resulting in the largest expected effect of the COVID-19 pandemic on disease outcomes. The first wave of COID-19 provided a unique circumstance for research. Further research during other COVID-19 waves and in other health care settings is encouraged.

## Conclusion

A slight decrease of patients presenting with acute appendicitis was found during the first wave of COVID-19 compared to a corresponding period in 2019. A decrease of uncomplicated cases was observed, while the proportion of complicated cases increased. This increase cannot only be explained by the increased prehospital delay during the COVID-19 pandemic, nor can it merely be explained by progression of uncomplicated to complicated appendicitis over time. More likely, part of the patients with mild, uncomplicated appendicitis may have resolved spontaneously at home, which is in line with the theory that uncomplicated and complicated appendicitis are different diseases and not simply different grades of severity.

## Supplementary Information

**Additional file 1: Figure S1.** Duration of symptoms at presentation, cohort 2019 versus 2020. **Table S1.** Comparison of patients with uncomplicated appendicitis for 2019 pre-COVID cohort vs. 2020 COVID cohort. **Table S2.** Comparison of patients with complicated appendicitis and with symptoms for more than 24 h, 2019 pre-COVID cohort versus 2020 COVID cohort. **Table S3.** Comparison of patients with an age of 60 years or higher of the 2019 pre-COVID cohort and 2020 COVID cohort. **Table S4.** Comparison of conservatively treated patients with appendicitis, 2019 pre-COVID cohort versus 2020 COVID cohort. **Table S5.** Comparison of conservatively treated patients by final (imaging) diagnosis and imaging modality, 2019 pre-COVID cohort versus 2020 COVID cohort. **Table S6.** COVID-19 positive appendicitis patients.

## Data Availability

The datasets generated and analyzed during the current study are not publicly available due to possible compromising individual privacy, but are available from the corresponding author on reasonable request.

## Abbreviations

CI: Confidence interval
COVID-19: Coronavirus disease 2019
CT: Computed tomography
ED: Emergency department
ICD-10: International Statistical Classification of diseases and related health problems, 10th edition
OR: Odds ratio
SARS-CoV-2: Severe Acute Respiratory Syndrome - Coronavirus-2
SPSS: Statistical Package for the Social Sciences, IBM software analytics
US: Ultrasound
