# Supplementary material for: Impact of the COVID-19 pandemic on incidence and severity of acute appendicitis: a comparison between 2019 and 2020
Source: BMC Emerg Med. 2021 May 12;21:61. doi: 10.1186/s12873-021-00454-y (PMC8114672; doi:10.1186/s12873-021-00454-y)
Supplement: Supplementary file 1 — Additional file 1: Figure S1. Duration of symptoms at presentation, cohort 2019 versus 2020. Table S1. Comparison of patients with uncomplicated appendicitis for 2019 pre-COVID cohort vs. 2020 COVID cohort. Table S2. Comparison of patients with complicated appendicitis and with symptoms for more than 24 h, 2019 pre-COVID cohort versus 2020 COVID cohort. Table S3. Comparison of patients with an age of 60 years or higher of the 2019 pre-COVID cohort and 2020 COVID cohort. Table S4. Comparison of conservatively treated patients with appendicitis, 2019 pre-COVID cohort versus 2020 COVID cohort. Table S5. Comparison of conservatively treated patients by final (imaging) diagnosis and imaging modality, 2019 pre-COVID cohort versus 2020 COVID cohort. Table S6. COVID-19 positive appendicitis patients. [file 12873_2021_454_MOESM1_ESM.docx]

**Impact of the COVID-19 pandemic on incidence and severity of acute appendicitis: a comparison between 2019 and 2020**

*Supplementary Material*

1. List of SCOUT investigators and affiliations page 2
2. Author Contributions  page 5
3. Figure S1 page 6
4. Table S1 page 7
5. Table S2 page 8
6. Table S3 page 9
7. Table S4 page 10
8. Table S5 page 11
9. Table S6 page 12
10. **List of SCOUT investigators and affiliations**

Authors

J.C.G. Scheijmans, MD^1^*, A.B.J. Borgstein, MD^2^*, C.A.J. Puylaert, MD, PhD^3^, W.J. Bom, MD^1^, S. Bachiri, MD^4^, E.A. van Bodegraven, MD^5^, A.T.A. Brandsma, MD^6^, F.M. ter Brugge, MD^7^, S.M.M. de Castro, MD, PhD^8^, R. Couvreur, MD^9^, L.C. Franken, MD, PhD^10^, M.P. Gaspersz, MD, PhD^11^, M.R. de Graaff, MD^12^, H. Groenen, MD^13^, S.C. Kleipool, MD^8^, T.J.L. Kuypers, MD^14^, M.H. Martens, MD, PhD^15^, D.M. Mens, BSc^16^, R.G. Orsini, MD, PhD^17^, N.J.M.M. Reneerkens, MD^18^, T. Schok, MD^19^, W.J.A. Sedee, MD^20^, S. Tavakoli Rad, MD^21^, J.H. Volders, MD, PhD^22^, P.D. Weeder, MD^23^, J.M. Prins, MD, PhD^24#^, H.A. Gietema, MD, PhD^25#^, J. Stoker, MD, PhD^26#^, S.S. Gisbertz, MD, PhD^2#^, M.G.H. Besselink, MD, PhD^2#^, M.A. Boermeester, MD, PhD^1#^, on behalf of the SCOUT study group

* Shared first authorship; these authors contributed equally to this article

^#^ Shared last authorship; these authors contributed equally to this article

Members of the SCOUT Collaborative study group

C.S. Andeweg, MD, PhD^27^, E.G. Boerma, MD, PhD^15^, M.D.M. Bolmers, MD^18^, M.J. Bolster-van Eenennaam, MD, PhD^12^, L.S.F. Boogerd, MD, PhD^18^, P. van Duijvendijk, MD, PhD^12^, S.L. Gans, MD, PhD^12^, A.A.W. van Geloven, MD, PhD^13^, P.D. Gobardhan, MD, PhD^16^, E.R. Hendriks, MD^13^, J.T. ten Holder, MD^28^, J.M. Hoogendoorn, MD, PhD^9^, T. de Hoop, MD^12^, J. van Kesteren, MD^18^, J.L.M. Konsten, MD, PhD^19^, S. Kucukcelebi, BSc^10^, A.M.F. Lopes Cardozo, MD^4^, G.M.H. Marres, MD, PhD^5^, R.A. Matthijsen, MD, PhD^14^, A.W.F. du Mée, MD^29^, S. van der Meij, MD, PhD^10^, J. Melenhorst, MD, PhD^17^, F.H.M. van Osch, PhD^30^, G.A. Patijn, MD, PhD^6^, R.A.W. Ploumen, MD^17^, T.J.M. Quanjel, MD^5^, T.F.D. van Rees Vellinga, MD^31^, M.A.J. de Roos, MD, PhD^22^, C.C. van Rossem, MD, PhD^11^, H.M.E. Quarles van Ufford, MD, PhD^31^, H.C. van Santvoort, MD, PhD^21,32^, N. Sosef, MD^23^, D. Schweitzer, MD^15^, T. Verhagen, MD^7^.

**Affiliations**

1. Department of Surgery, Amsterdam UMC, location AMC, Amsterdam Gastroenterology Endocrinology Metabolism, University of Amsterdam, Amsterdam, The Netherlands
2. Department of Surgery, Cancer Center Amsterdam, Amsterdam UMC, University of Amsterdam, Amsterdam, the Netherlands
3. Department of Radiology and Nuclear Medicine, Amsterdam Gastroenterology Endocrinology Metabolism, Amsterdam UMC, University of Amsterdam, Amsterdam, the Netherlands
4. Department of Surgery, Noordwest Hospital Group, Alkmaar, the Netherlands
5. Department of Surgery, Albert Schweitzer Hospital, Dordrecht, the Netherlands
6. Department of Surgery, Isala Hospital, Zwolle, the Netherlands
7. Department of Surgery, Hospital Group Twente, Almelo, the Netherlands
8. Department of Surgery, OLVG, Amsterdam, the Netherlands
9. Department of Surgery, Haaglanden Medical Center, The Hague, the Netherlands
10. Departement of Surgery, Flevo Hospital, Almere, the Netherlands
11. Department of Surgery, Maasstad Hospital, Rotterdam, the Netherlands
12. Department of Surgery, Gelre Hospitals, Apeldoorn, the Netherlands
13. Department of Surgery, Tergooi Hospitals, Hilversum, the Netherlands
14. Department of Surgery, Elisabeth - Tweesteden Hospital, Tilburg, the Netherlands
15. Department of Surgery, Zuyderland Medical Center, Sittard-Geleen/Heerlen, the Netherlands
16. Department of Surgery, Amphia Hospital, Breda, the Netherlands
17. Department of Surgery, Maastricht UMC+, Maastricht, the Netherlands
18. Department of Surgery, Dijklander Hospital, Hoorn, the Netherlands
19. Department of Surgery, VieCuri Medisch Centrum for Noord-Limburg, Venlo, the Netherlands
20. Department of Emergency Medicine, St Jansdal Hospital, Harderwijk, the Netherlands
21. Department of Surgery, Sint Antonius Hospital, Nieuwegein, the Netherlands
22. Department of Surgery, Rijnstate Hospital, Arnhem, the Netherlands
23. Department of Surgery, Spaarne Gasthuis, Haarlem and Hoofddorp, the Netherlands
24. Department of Internal Medicine, Division of Infectious Diseases, Amsterdam Institute for Infection and Immunity (AI&II), Amsterdam UMC, University of Amsterdam, Amsterdam, the Netherlands
25. Department of Radiology and Nuclear Medicine, Maastricht UMC+, Maastricht, the Netherlands
26. Department of Radiology and Nuclear Medicine, Amsterdam Gastroenterology Endocrinology Metabolism, Amsterdam UMC, University of Amsterdam, Amsterdam, the Netherlands
27. Department of Surgery, St Jansdal Hospital, Harderwijk, the Netherlands
28. Department of Pulmonary Medicine, Haaglanden Medical Center, The Hague, the Netherlands
29. Department of Radiology, Amphia Hospital, Breda, the Netherlands
30. Department of Clinical Epidemiology, VieCuri Medisch Centrum for Noord-Limburg, Venlo, the Netherlands
31. Department of Radiology and Nuclear Medicine, Haaglanden Medical Center, The Hague, the Netherlands
32. Department of Surgery, UMC Utrecht Cancer Center, UMC Utrecht, the Netherlands
33. **Author Contributions**

*Concept and design:* Scheijmans, Borgstein, Puylaert, Bom, Prins, Gietema, Stoker, Gisbertz, Besselink and Boermeester.

*Acquisition of data:* Bachiri, van Bodegraven, Brandsma, ter Brugge, de Castro, Couvreur, Franken, Gaspersz, de Graaff, Groenen, Kleipool, Kuypers, Martens, Mens, Orsini, Reneerkens, Schok, Sedee, Tavakoli Rad, Volders, Weeder.

*Analysis and interpretation of data:* Scheijmans, Borgstein, Puylaert, Bom, Prins, Gietema, Stoker, Gisbertz, Besselink and Boermeester.

*Statistical analysis:* Scheijmans, Borgstein.

*Drafting of the manuscript:* Scheijmans, Borgstein, Puylaert, Bom, Prins, Gietema, Stoker, Gisbertz, Besselink and Boermeester.

*Critical revision of the manuscript:* All authors.

*Final approval of the manuscript for publication:* All authors.

1. **Figure S1.** Duration of symptoms at presentation, cohort 2019 versus 2020.

1. **Table S1.** Comparison of patients with uncomplicated appendicitis for 2019 pre-COVID cohort vs. 2020 COVID cohort.

| **Characteristic** | **2019 control cohort (n=391)** | **2020 COVID-19 cohort**  **(n=321)** | P value |
| --- | --- | --- | --- |
| Age, median (IQR), years | 36 (26-52) | 37 (28-51) | 0.227 |
| ASA >1, no./total no. (%) | 136/364 (37.4) | 121/282 (42.9) | 0.153 |
| Duration of symptoms >24 hours, no./total no. (%) | 201/390 (51.5) | 163/317 (51.4) | 0.975 |
| Conservative treatment, no./total no. (%) | 10/391 (2.6) | 22/321 (6.9) | **0.006** |
| In-hospital delay in operated patients, median (IQR), hours | 7.0 (4.8-14.1) | 6.3 (4.1-10.7) | **0.003** |
| Postoperative complication*, no./total no. (%) | 28/381 (7.3) | 18/298 (6.0) | 0.501 |
| Severe postoperative complication*°, no./total no. (%) | 4/381 (1.0) | 7/298 (2.3) | 0.227^¥^ |
| Abbreviations: ASA, American Society of Anesthesiologists; IQR, interquartile range.  ¥ Fischer exact test was performed  * Patients for whom surgery was the initial treatment.  ○ Severe complications are defined as Clavien-Dindo IIIa or higher. | | | |

1. **Table S2.** Comparison of patients with complicated appendicitis and with symptoms for more than 24 hours, 2019 pre-COVID cohort versus 2020 COVID cohort

| **Characteristic** | **Cohort 2019 (n=164)** | **Cohort 2020 (n=214)** | P value |
| --- | --- | --- | --- |
| Age, median (IQR), years | 49 (32-65) | 52 (33-65) | 0.689 |
| Female sex, no./total no. (%) | 64/164 (39.0) | 101/214 (47.2) | 0.112 |
| ASA >1, no./total no. (%) | 81/147 (55.1) | 91/177 (51.4) | 0.508 |
| Severity of appendicitis, no./total no. (%) |  |  |  |
| Gangrenous | 19/164 (11.6) | 32/214 (15.0) | 0.342 |
| Perforation | 109/164 (66.5) | 137/214 (64.0) | 0.621 |
| Abscess or infiltrate | 36/164 (22.0) | 45/214 (21.0) | 0.828 |
| Complications within 30 days, no./total no. (%) | 25/164 (15.2) | 46/214 (21.5) | 0.123 |
| Abbreviations: ASA, American Society of Anesthesiologists; IQR, interquartile range. | | | |

| **Characteristic** | **Cohort 2019 (n=138)** | **Cohort 2020 (n=136)** | P value |
| --- | --- | --- | --- |
| Female sex, no./total no. (%) | 64/138 (46.4) | 72/136 (52.9) | 0.277 |
| ASA >1, no./total no. (%) | 111/130 (85.4) | 95/105 (90.5) | 0.238 |
| Duration of symptoms >24 hours, no./total no. (%) | 82/136 (60.3) | 96/133 (72.2) | **0.039** |
| Severity of appendicitis, no./total no. (%) |  |  | 0.132^¥^ |
| Uncomplicated | 57/138 (41.3) | 42/136 (31.6) |  |
| Gangrenous | 11/138 (8.0) | 5/136 (3.7) |  |
| Perforation | 56/138 (40.6) | 68/136 (50.0) |  |
| Abscess or infiltrate | 13/138 (9.4) | 19/136 (14.0) |  |
| Complications within 30 days, no./total no. (%) | 27/138 (19.6) | 26/136 (191.1) | 0.925 |
| **Patients older than 60 years, presented with durations of symptoms >24 hours** | | | |
|  | N=82 | N=96 |  |
| Complications within 30 days, no./total no. (%) | 18/82 (22.0) | 20/96 (20.8) | 0.856 |
| Abbreviation: ASA, American Society of Anesthesiologists  ¥ Fischer exact test was performed | | | |

1. **Table S3.** Comparison of patients with an age of 60 years or higher of the 2019 pre-COVID cohort and 2020 COVID cohort.
2. **Table S4.** Comparison of conservatively treated patients with appendicitis, 2019 pre-COVID cohort versus 2020 COVID cohort

| **Characteristic** | **Cohort 2019 (n=41)** | **Cohort 2020 (n=63)** | P value |
| --- | --- | --- | --- |
| Severity of appendicitis based on imaging, no./total no. (%) |  |  | 0.339^¥^ |
| Uncomplicated | 10/41 (24.4) | 22/63 (34.9) |  |
| Gangrenous | 0/41 (0) | 0/63 (0) |  |
| Perforation | 2/41 (4.9) | 6/63 (9.5) |  |
| Abscess or infiltrate | 29/41 (70.7) | 35/63 (55.6) |  |
| Radiological drainage (initial treatment), no./total no. (%) | 6/41 (14.6) | 11/63 (17.5) | 0.873^¥^ |
| Complication during the first 30 days, no./total no. (%) | 3/41 (7.3) | 11/62 (17.7) | 0.154^¥^ |
| Appendectomy within 30 days, no./total no. (%) | 4/41 (9.8) | 11/63 (17.5) | 0.394^¥^ |
| Severity of appendicitis based on appendectomy within 30 days, no./total no. (%) |  |  | 0.506^¥^ |
| Uncomplicated | 3/4 (75) | 3/11 (27.3) |  |
| Gangrenous | 0/4 (0) | 1/11 (9.1) |  |
| Perforation | 0/4 (0) | 4/11 (36.4) |  |
| Abscess or infiltrate | 1/4 (25) | 2/11 (18.2) |  |
| Postoperative complication*, no./total no. (%) | 0/4 (0) | 3/10 (30.0) | 0.505^¥^ |
| Severe postoperative complication*°, no./total no. (%) | 0/4 (0) | 1/10 (10.0) | 1.000^¥^ |
| ¥ Fischer exact test was performed  * Patients for whom surgery was the initial treatment.  ○ Severe complications are defined as Clavien-Dindo IIIa or higher. | | | |

1. **Table S5.** Comparison of conservatively treated patients by final (imaging) diagnosis and imaging modality, 2019 pre-COVID cohort versus 2020 COVID cohort

| **Characteristic** | **2019 control cohort** | | | **2020 COVID-19 cohort** | | |
| --- | --- | --- | --- | --- | --- | --- |
|  | Uncomplicated appendicitis | Complicated appendicitis | | Uncomplicated appendicitis | Complicated appendicitis | |
|  |  | Perforated | Infiltrate/abscess |  | Perforated | Infiltrate/abscess |
| US only | 4 | 1 | 11 | 6 | 0 | 4 |
| CT only | 4 | 1 | 4 | 9 | 5 | 19 |
| US + CT/MRI* | 2 | 0 | 14 | 7 | 0 | 11* |
|  | 10 | 31 (76%) | | 22 | 39 (64%) | |
| Total | 41 | | | 61 | | |
| Abbreviations: CT, computed tomography; MRI, magnetic resonance imaging; US, ultrasound.  * Only one patient was diagnosed by MRI after US. | | | | | | |

1. **Table S6.** COVID-19 positive appendicitis patients.

| **COVID-19 positive** | **Cohort 2020 (n=12)** |
| --- | --- |
| Severity of appendicitis based on imaging, no./total no. (%) |  |
| Uncomplicated | 3/12 (25) |
| Gangrenous | 0/12 (0) |
| Perforation | 8/12 (66.7) |
| Abscess or infiltrate | 1/12 (8.3) |
| Conservative treatment, no./total no. (%) | 3/12 (25) |
| COVID-19 diagnosis based on |  |
| RT-PCR | 11/12 (91.7) |
| CT | 1/12 (8.3) |
